# Roles of Aryl Hydrocarbon Receptor in Aromatase-Dependent Cell Proliferation in Human Osteoblasts

**DOI:** 10.3390/ijms18102159

**Published:** 2017-10-17

**Authors:** Yasuhiro Miki, Shuko Hata, Katsuhiko Ono, Takashi Suzuki, Kiyoshi Ito, Hiroyuki Kumamoto, Hironobu Sasano

**Affiliations:** 1 Department of Disaster Obstetrics and Gynecology, International Research Institute of Disaster Science (IRIDeS), Tohoku University, Sendai, Miyagi 980-8575, Japan; kito@med.tohoku.ac.jp; 2 Department of Anatomic Pathology, Tohoku University Graduate School of Medicine, Sendai, Miyagi 980-8575, Japan; hatashu@med.tohoku.ac.jp (S.H.); k-ono@patholo2.med.tohoku.ac.jp (K.O.); hsasano@patholo2.med.tohoku.ac.jp (H.S.); 3 Department of Oral Pathology, Tohoku University Graduate School of Dentistry, Sendai, Miyagi 980-8575, Japan; kumamoto@m.tohoku.ac.jp; 4 Department of Pathology and Histotechnology, Tohoku University Graduate School of Medicine, Sendai, Miyagi 980-8575, Japan; t-suzuki@patholo2.med.tohoku.ac.jp

**Keywords:** aryl hydrocarbon receptor, aromatase, osteoblast

## Abstract

Aryl hydrocarbon receptor (AhR) is a ligand-activated transcription factor and its expression is influenced by environmental compounds, such as 3-methylcholanthrene (3-MC) and β-naphthoflavone (β-NF). AhR and its downstream genes, such as *CYP1A1*, are considered to play a pivotal role in xenobiotic responses. AhR signaling has also been proposed to mediate osteogenesis in experimental animals, but its details have remained unclear. Therefore, in this study, we examined the possible roles of AhR in human bone. Immunohistochemical analysis revealed that AhR was detected in both osteoblasts and osteoclasts. We then screened AhR-target genes using a microarray analysis in human osteoblastic hFOB cells. Results of microarray and subsequent PCR analysis did reveal that estrogen metabolizing and synthesizing enzymes, such as CYP1B1 and aromatase, were increased by 3-MC in hFOB and osteosarcoma cell line, MG-63. The subsequent antibody cytokine analysis also demonstrated that interleukin-1β and -6 expression was increased by 3-MC and β-NF in hFOB cells and these interleukins were well known to induce aromatase. We then examined the cell proliferation rate of hFOB and MG-63 cells co-treated with 3-MC and testosterone as an aromatase substrate. The status of cell proliferation in both hFOB and MG-63 cells was stimulated by 3-MC and testosterone treatment, which was also inhibited by an estrogen blocker, aromatase inhibitor, or AhR antagonist. These findings indicated that AhR could regulate estrogen synthesis and metabolism in bone tissues through cytokine/aromatase signaling.

## 1. Introduction

Endocrine disrupting chemicals (EDCs) are well-known to affect the skeletal system as teratogenic factors in animals. Recently, the direct actions and molecular mechanisms of EDCs in bone cells have been studied [1]. Alkylphenols, such as nonylphenol and octylphenol, inhibit the formation of osteoclasts in co-cultures of murine osteoclast precursor cells with osteoclast-supporting cells [1]. EDCs also exert various effects on bone cells through nuclear receptors, such as the estrogen receptor (ER) [2], peroxisome proliferator-activated receptor γ (PPAR γ) [3], and steroid and xenobiotic receptor (SXR) [4]. Among these receptors, aryl hydrocarbon receptor (AhR) plays pivotal roles in mediating the action of EDCs. AhR is a ligand-activated transcription factor activated by various EDCs, including 3-methylcholanthrene (3-MC) and tetrachlorodibenzodioxin (TCDD) [5,6]. AhR is also activated by ligand binding to mediate transcriptional activation of cytochrome P450 (CYP) 1A, CYP1B, etc. [5,6,7]. Therefore, AhR and its downstream pathways were reported to play a pivotal role as a xenobiotic sensor in lung and liver tissues [5,6,7]. AhR is also reported in mesenchymal stem cell-derived cells, such as adipocytes [8], myofibroblasts [9], and fibroblasts [10]. In bone cells, direct anti-proliferative or anti-osteogenesis effects of AhR ligands, such as 3-MC and TCDD, have also been reported [5,11,12,13,14,15].

Aromatase (CYP19A) plays a crucial role in intracrine estrogen synthesis and has been considered a major target molecule for endocrine disruption [16,17,18]. Aromatase converts androgens (androstenedione and testosterone) into estrogens (estrone and androstenedione). Aromatase was also previously reported to be present in human bone tissues, especially osteoblasts [19], and to be involved in estrogen-dependent cell proliferation of osteoblasts in vitro [20]. AhR agonists, such as TCDD and β-naphthoflavone (β-NF) were also reported to exert inhibitory effects on aromatase expression in the granulosa cell line, KGN [16]. In addition, in the human hepatoma HepaRG cell line, the aromatase gene was reported to be up-regulated by β-NF treatment [17]. Polychlorinated biphenyl 153, phthalate, and bisphenol A induced aromatase gene expression in breast cancer cells and endometriosis tissues [18]. We recently demonstrated that the treatment with TCDD significantly induced aromatase mRNA expression in breast carcinoma cell lines MCF-7, T-47D, and MDA-MB-231 [21]. In addition, in breast carcinoma tissues, the H score of aromatase was significantly higher in AhR-positive areas than negative areas of the tumor [21]. These findings did indicate that the regulation of aromatase expression caused by AhR agonists could possibly include organ- or tissue-specificity. However, the effects of EDCs on aromatase regulation by AhR activation have remained totally unknown in human osteoblasts, an important target of EDCs.

Therefore, in this study, we first immunolocalized AhR protein in human bone tissues. We then screened the expression of AhR inducible factors using an oligonucleotide microarray in a human osteoblast cell line (hFOB) treated with 3-MC. We, subsequently, examined the aromatase induction by treatment with AhR agonists in both hFOB and osteoblast-like MG-63 cell lines. We also identified the expression of aromatase-inducible cytokines released from hFOB cells using an anti-cytokine antibody array in order to further explore the correlation between AhR and estrogen biosynthesis in human osteoblasts.

## 2. Results

### 2.1. Expression of AhR in Human Bone Tissues

**Figure 1:**
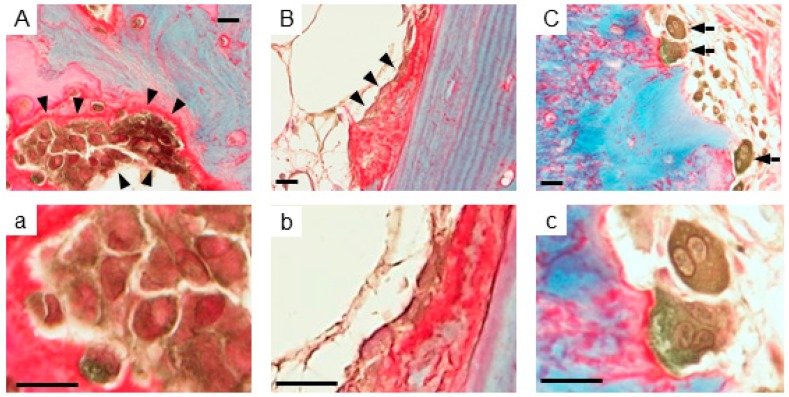

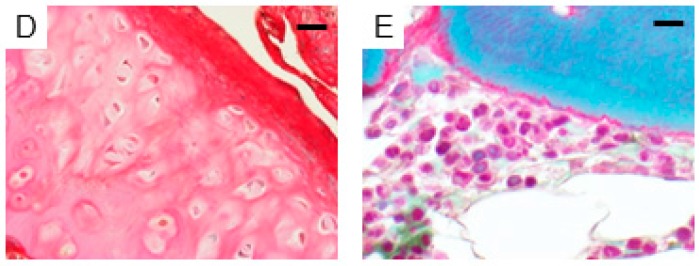
Immunohistochemistry of AhR in bone tissues. (**A**,**B**). AhR immunoreactivity (**brown**) was detected in osteoblasts (**arrowheads**); (**a**) and (**b**) are high magnifications of (**A**) and (**B**), respectively. AhR was detected at high levels in the cytoplasm (**brown**) and low levels in nuclei (**reddish-brown**). (**C**). AhR immunoreactivity (**brown**) was detected in osteoclasts (**arrows**); (**c**) is a high magnification of (**C**). Relatively high levels of AhR immunoreactivity were detected in both cytoplasm and nucleus. No AhR immunoreactivity was detected in (**D**) chondrocytes and (**E**) bone stromal cells. Collagen protein was stained red. Non-collagen proteins including calcified bone matrix were stained bluish-green. Both cytoplasm and nuclei were stained red using the K61 Collagen Stain Kit (Collagen Research Center, Kiyose, Tokyo, Japan). Scale bar, 5 μm. AhR, aryl hydrocarbon receptor.

AhR immunoreactivity was detected in the cytoplasm of epithelial cells on the bronchiole (data not shown). In bone (Figure 1), AhR immunoreactivity was detected in both the nuclei and cytoplasm of osteoblasts, which were located on the collagen-positive (red stained area) osteoid surface (Figure 1(Aa,Bb)). Osteoclasts, multinucleated cells in bone resorption cavities, were also positive for AhR (Figure 1Cc). There was no AhR immunoreactivity in chondrocytes (Figure 1D) and bone stromal cells (Figure 1E), respectively.

### 2.2. Characteristics of Osteoblast and Osteosarcoma Cell Lines Used in This Study

**Figure 2:**
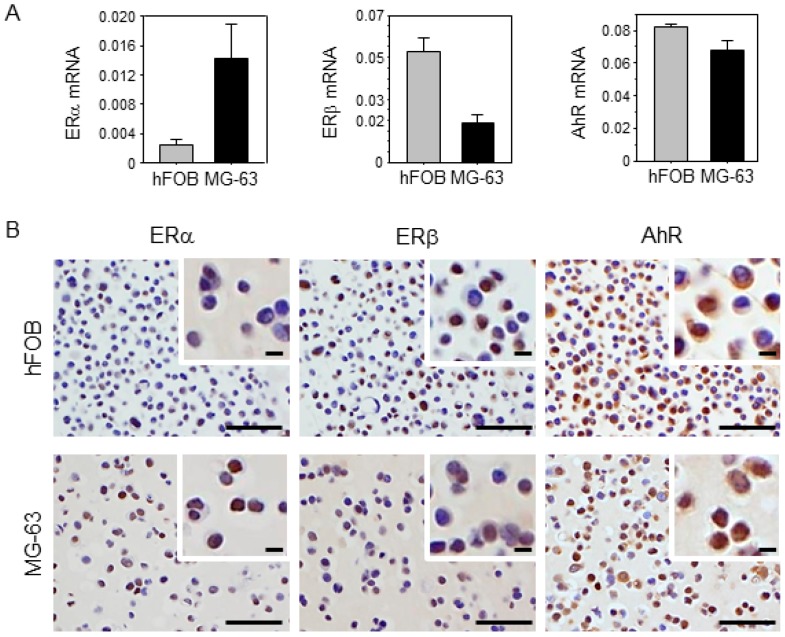
Characteristics of osteoblast (hFOB) and osteosarcoma (MG-63) cell lines. (**A**) Expression levels of ERα, ERβ, and AhR in hFOB and MG-63 cells. Data are presented as mean and standard deviation (*n* = 3). (**B**) Immunocytochemistry of ERα, ERβ, and AhR in hFOB and MG-63 cells. ERα and ERβ immunoreactivities in nuclei were predominantly detected in MG-63 and hFOB cells, respectively. AhR immunoreactivity was detected in both cytoplasm and nuclei in hFOB and MG-63 cells. Scale bar, 50 μm. Upper-right areas are high magnifications of each image. Scale bar, 5 μm.

Expression levels of ERα, ERβ, and AhR are shown in Figure 2. High levels of ERα and ERβ were detected in hFOB and MG-63 cell lines, respectively, by real-time PCR. The AhR expression level was similar in hFOB and MG-63 cells. Relatively high levels of ERα and ERβ immunoreactivities were detected in hFOB and MG-63 cells, respectively. AhR immunoreactivity was detected in both cytoplasm and nuclei of hFOB and MG-63 cells.

### 2.3. Gene Expression Induced by 3-MC Treatment in hFOB

The genes induced by 3-MC in hFOB cells were summarized in Table 1. *COL18A1* was most increased compared to the control among those examined. The aromatase gene (*CYP19A1*) had 3.2-fold higher expression following 3-MC treatment. The steroid hormone-related genes, such as *ESR2*, *CYP21A2*, and *CYP1B1*, were also increased by 3-MC treatment.

### 2.4. Effects of AhR Agonists on Aromatase Expression in Osteoblasts

**Figure 3:**
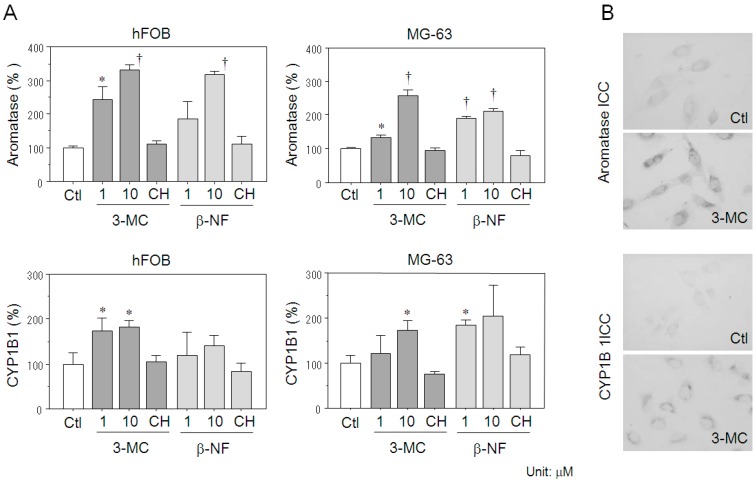
Induction of aromatase and CYP1B1 by AhR ligands in osteoblasts. (**A**) Both 3-methylcholanthrene (3-MC) and β-naphthoflavone (β-NF) significantly increased aromatase expression in hFOB and MG-63 cells. There were no significant changes in aromatase expression in both hFOB and MG-63 cells co-treated with AhR agonist (3-MC or β-NF) and its antagonist (CH-223191) compared to vehicle control cells. * *p* < 0.05; ^†^ *p* < 0.01; Ctl, vehicle control (0.005% dimethyl sulfoxide); CH, 10 μM CH-223191. (**B**) 3-MC markedly induced expression of both aromatase and CYP1B1 immunoreactivities compared with vehicle control in hFOB cells. ICC, immunocytochemistry; Ctl, vehicle control (0.005% dimethyl sulfoxide); 3-MC, 10 μM 3-methylcholanthrene. Data are presented as mean and standard deviation (*n* = 3).

In hFOB cells, both 3-MC (1 μM (*p* = 0.0026) and 10 μM (*p* < 0.0001)) and β-NF (10 μM (*p* < 0.0001)) significantly increased aromatase mRNA levels (Figure 3A). Aromatase mRNA levels in MG-63 cells were also increased by treatment of both 3-MC (1 μM (*p* = 0.0030) and 10 μM (*p* < 0.0001)) and β-NF (1 μM (*p* < 0.0001) and 10 μM (*p* < 0.0001)) (Figure 3A). The increase of aromatase expression by both 3-MC (10 μM) and β-NF (10 μM) were suppressed to the control level by co-treatment with the AhR antagonist, CH-223191, in both hFOB and MG-63 cells. There were no significant changes in hFOB and MG-63 cells treated with 2 and 10 μM AhR antagonist alone. Treatment with 50 μM AhR antagonist demonstrated a cytotoxic effect in both hFOB and MG-63 cells. Therefore, in this study, we employed 10 μM AhR antagonist for the subsequent experiments. In hFOB cells, CYP1B1 mRNA level was also significantly increased by 3-MC treatment (1 μM (*p* = 0.0298) and 10 μM (*p* = 0.0076)), and these increments were significantly inhibited by 10 μM CH-223191 co-treatment. There were no significant changes of CYP1B1 mRNA expression in hFOB cells treated with β-NF. In MG-63 cells, both 3-MC (10 μM (*p* = 0.0114)) and β-NF (1 μM (*p* = 0.0019)) significantly increased aromatase mRNA levels (Figure 3A). The increment of CYP1B1 expression described above was also significantly suppressed to control levels by CH-223191 co-treatment.

Immunocytochemical analysis revealed that both aromatase and CYP1B1 were not detected in control hFOB cells, whereas they were detected following 3-MC (10 μM) treatment (Figure 3B).

### 2.5. Cytokines Secreted from hFOB Cells Stimulated by AhR Agonists

**Figure 4:**
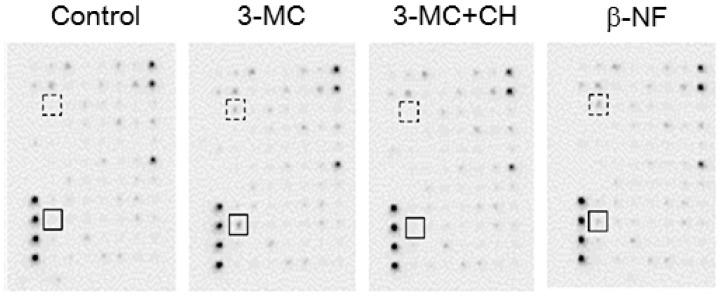
Cytokine profiles derived from hFOB cells treated with AhR agonists. Both IL-6 and IL-1β immunoreactivities were detected in hFOB cells treated with AhR agonists (10 μM 3-MC and 10 μM β-NF). Both IL-6 and IL-1β were not expressed at detectable levels in hFOB cells co-treated with AhR antagonist and 3-MC as well as in control cells (vehicle control; 0.005% dimethyl sulfoxide). Solid line square, IL-6; dashed line square, IL-1β; CH, 10 μM CH-223191.

Interleukin (IL)-1β and IL-6 immunoreactivity was detected in 3-MC-treated hFOB cells, but not in control hFOB cells (Figure 4). The immunoreactivity was inhibited by co-treatment of CH-223191 with 3-MC. Both IL-1β and IL-6 were also detected in hFOB cells treated with β-NF. There were no significant differences of other cytokines as the results of cytokine antibody array in hFOB cells treated with either 3-MC or β-NF.

### 2.6. Estrogen-Dependent Cell Proliferation in Osteoblasts through the 3-MC-Induced Aromatase Pathway

**Figure 5:**
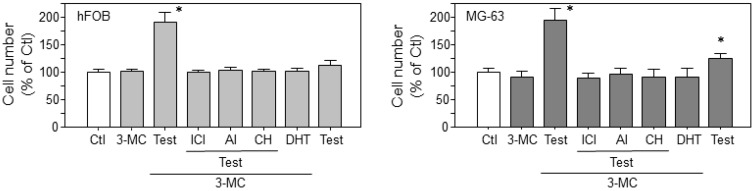
Cell proliferation effects of AhR agonists through the aromatase pathway. Co-treatment with testosterone and 3-MC (10 μM) significantly increased cell numbers of both hFOB and MG-63 cells. * *p* < 0.05; Ctl, vehicle control (0.005% dimethyl sulfoxide); 3-MC, 10 μM 3-methylcholanthrene; BPA; Test, 10 nM testosterone; ICI, 10 μM ICI 182,780; AI, 10 μM Aromatase Inhibitor I; CH, 10 μM CH-223191; DHT, 10 nM dihydrotestosterone. Data are presented as mean and standard deviation (*n* = 6).

The co-treatment with testosterone, the endogenous aromatase substrate, and 3-MC significantly increased the cell numbers in both hFOB (*p* < 0.0001) and MG-63 (*p* < 0.0001) cells compared to those in controls (Figure 5). This increased cell number was significantly inhibited by co-treatment with estrogen blocker (ICI 182,780), aromatase inhibitor (Aromatase inhibitor I), or AhR antagonist (CH-223191). The co-treatment with 3-MC and dihydrotestosterone (DHT) exerted no effects upon the cell numbers of either hFOB or MG-63 cells. Treatment with testosterone alone significantly increased the number of MG-63 cells (*p* = 0.0004), but not of hFOB cells.

## 3. Discussion

Aromatase plays a pivotal role in estrogen synthesis in normal and cancerous tissues. In osteoblasts, aromatase activation did increase the number of cells in vitro and bone mass in vivo resulting from increased local estrogen concentration [22]. CYP1B1 catalyzes the 4-hydroxylation of estrogens [23] and, therefore, could possibly attenuate estrogenic actions. In this study, AhR ligands induced both aromatase and CYP1B1 genes in both osteoblast and osteoblast-like cells. Testosterone treatment significantly increased the cell proliferation rate in both hFOB and MG-63 cells stimulated by 3-MC treatment. In addition, the number of the cells increased above was significantly inhibited by the treatment with an aromatase inhibitor, ER blocker, or AhR antagonist. In our previous study, TCDD did induce both aromatase and CYP1A1 via binding to AhR in breast carcinoma cell lines [21]. CYP1A1 is well known as a target gene for AhR signaling and catalyzes estrogen conversion [21]. Aromatase-dependent cell proliferation was also detected in breast carcinoma cell lines stimulated by TCDD treatment [21]. These findings all indicated that estrogen synthesis by aromatase could surpass the estrogen metabolism or degradation through CYP1B1 in response to the AhR mediated intracellular signals activated by EDCs in osteoblasts. CYP1B1 polymorphisms were also reported to alter bone density [24,25]. However, the CYP1B1 expression patterns in human bone tissues have not been reported at all, to the best of our knowledge. In addition, 4-hydroxyestradiol produced by CYP1B1 enzymatic activities resulted in carcinogenesis through free radical generation [26]. Therefore, further functional analyses of CYP1B1 are required to clarify the possible roles of AhR-mediated intracellular signaling in endocrine-disrupting effects of human bone tissues.

Osteoblasts secrete several types of inflammatory cytokines including ILs. Results of cytokine antibody array analysis in our present study did reveal that both IL-1β and IL-6, both of which are well known to be up-regulated by AhR signaling [27,28], were increased by AhR agonists in hFOB cells. Therefore, in this study, we focused upon the effects of IL-1β and IL-6 secretion in response to AhR activation as a mechanism of aromatase gene induction in osteoblasts in vitro. Both IL-1β and IL-6 are well known to up-regulate the aromatase gene transcripts and its activity in several types of cells, including osteoblasts (IL-1β) [29] and fibroblasts derived from lung cancer (IL-6) [30]. AhR binds to a xenobiotic response element (XRE) located in the promoter region of target genes and exerts transcriptional activation in these target genes [6]. Both IL-1β and IL-6 harbored XRE sequences in their promoter region similar to typical AhR target genes, such as CYP1A1 and CYP1B1 [6]. Therefore, results from our present study did indicate that EDCs, which activate AhR, could possibly disturb the intratissue estrogen balance by inducing aromatase through cytokines secreted by the AhR pathway. However, the function of XREs in the aromatase gene promoter region has remained unknown and, therefore, further examination regarding direct regulation of aromatase by AhR is required for clarifying the estrogen balance disruption caused by EDCs in human bone tissue.

ERβ is a predominant estrogen receptor in osteoblasts [31,32] and plays a pivotal role in estrogen-dependent cell proliferation in osteoblasts in vitro [32]. Many EDCs, especially phytoestrogens, are also well-known to bind to ERβ with higher affinity than to ERα [33]. Therefore, EDCs could possibly activate both ERβ and AhR disturb estrogen signaling by binding to ERβ, which is increased by AhR activation in osteoblasts. In addition, indirect crosstalks between ER and AhR signaling have been proposed [33]. Upon activation by its ligand, AhR was translocated from the cytoplasm into the nucleus using molecular chaperones, such as AhR nuclear translocator (ARNT) and regulated gene expression [6,34]. ARNT is a coactivator of both ERα and ERβ, but predominantly with ERβ [35,36]. Activated AhR recruits cofactors, such as RIP140, SRC-1, and SRC-2, which are also important in ER signaling [35,36]. These findings did indicate that activated AhR disturbed ER signaling, such as estrogen-dependent growth, by competing for common cofactors. However, in our present study, the number of cells was increased by 3-MC treatment through the ER in both hFOB and MG-63 cells. In breast carcinoma cells, the crosstalk between AhR and ER was recently reported to be transient and immediately disappeared following ligand removal [21]. However, the aromatase induction by the AhR signal is maintained even if the ligand is removed [21]. Therefore, the induction of the aromatase pathway by AhR activation is considered relatively continuous, whereas the crosstalk between ER and AhR could be temporal, but further investigations are required for clarification. In addition, 3-MC treatment increased ERβ gene (ESR2) expression in microarray analysis, but the validation of this increase by real-time PCR was not carried out.

Results of our present immunohistochemical analysis also revealed the absence of AhR in chondrocytes. Cedervall et al. reported the presence of AhR immunoreactivity in human growth plate cartilage [15], but in their study, the percentage of AhR-positive chondrocytes decreased in the order: hypertrophic zone > proliferative zone > resting zone of the human growth plate in boys and girls [15]. Therefore, AhR signaling could possibly play a crucial role in bone growth in children and adolescents. In addition, AhR immunoreactivity was also detected in osteoclasts. Several studies demonstrated the role of AhR signaling in osteoclastogenesis and osteoclast-dependent bone homeostasis [11,14,37]. Estrogen is well known to directly influence growth and differentiation of bone cells and bone remodeling through ER-mediated intracellular signaling [38,39]. Therefore, those findings and results of our present study did indicate that EDCs could possibly disturb bone cell homeostasis maintenance by disrupting the intracrine estrogen balance through AhR.

## 4. Materials and Methods

### 4.1. Immunohistochemistry and Histochemistry

Non-pathological adult bone tissues were retrieved from surgical pathology or autopsy files (two females and three males, 17 to 55 years old) from the Department of Pathology, Tohoku University Hospital (Sendai, Japan). The Ethics Committee at the Tohoku University School of Medicine approved the research protocols (no. 204-110, 2014). Tissue sections were immunostained using a biotin-streptavidin method and the Histofine kit (Nichirei, Tokyo, Japan). A rabbit polyclonal antibody against AhR was purchased from BIOMOL International (Farmingdale, NY, USA). Normal lung was used as a positive control for AhR [21].

Histochemical analysis of collagen was performed using the K61 Collagen Stain Kit (Collagen Research Center, Tokyo, Japan). The tissue specimens were incubated for 30 min at room temperature with a green dye (0.1% Fast Green FCF) that stains non-collagenous proteins and a red dye (0.1% Sirius Red F3B) that specifically stains collagen. The procedures of immunohistochemistry and histochemistry have been previously described in detail [4,21].

### 4.2. Chemicals

AhR agonists (3-MC and β-NF) and AhR antagonist (CH-223191) were purchased from Sigma-Aldrich (St. Louis, MO, USA). Testosterone and DHT were purchased from Wako Pure Chemical industries (Osaka, Japan). Aromatase inhibitor I [4-(Imidazolylmethyl)-1-nitro-9H-9-xanthenone] was purchased from Calbiochem (Merck, Darmstadt, Germany). Test materials were dissolved in dimethyl sulfoxide (DMSO; Wako Pure Chemical industries, Richmond, VA, USA). The final concentrations of DMSO used in this study did not exceed 0.005% in any of the cases examined.

### 4.3. Osteoblast and Osteosarcoma Cell Lines and Culture Conditions

The human osteoblast cell line, hFOB 1.19 (CRL-11372), was obtained from American Type Culture Collection (Manassas, VA, USA). The hFOB was maintained in a mixture of Dulbecco’s Modified Eagle Medium and Ham’s F12 medium (Invitrogen Life Technologies, Carlsbad, CA, USA) supplemented with 10% fetal bovine serum (FBS; JRH Biosciences, Lenexa, KS, USA) and 50 mg/mL G 418 sulfate (EMD Biosciences, San Diego, CA, USA) (passage number: 20 to 30). The human osteosarcoma cell line MG-63 was provided by the Cell Resource Center for Biomedical Research, Tohoku University (Sendai, Japan), and was maintained in RPMI-1640 medium (Sigma-Aldrich) (passage number: 30 to 40). The detailed culture conditions were reported in our previous studies [4,20,32]. The cells were pre-cultured for 48 h with 10% dextran-coated charcoal-stripped FBS (DCC-FBS) before examination. The cells were then harvested in DCC-FBS medium and were plated in 96-well plates or 100-mm culture dishes at an initial concentration of 3 × 10^4^ cells/mL.

### 4.4. Characteristics of Osteoblast and Osteosarcoma Cell Lines

Expressions of ERα, ERβ, and AhR were analyzed using real-time PCR and immunocytochemistry. The methodology of real-time PCR is described in Section 4.5. Real-Time PCR. For immunocytochemical analysis, cultured hFOB, and MG-63 cells were trypsinized and collected in 1.5-mL sample tubes. After centrifugation, cell pellets were fixed for 5 min in 10% neutral buffered formalin (Wako Pure Chemical) and congealed in iPGell (GenoStaff, Tokyo, Japan). iPGell clots were then embedded in paraffin. Antibodies employed in immunocytochemical analysis were as follows: ERα, mouse monoclonal 6F11 (Leica, Wetzlar, Germany); ERβ, mouse monoclonal 14C8 (GeneTex, Irvine, CA, USA); and AhR, rabbit polyclonal (BIOMOL) antibodies.

### 4.5. Microarray Analysis

hFOB cells were cultured in a 100-mm culture dish and treated with 10 μM 3-MC. hFOB cell lysates were prepared using RLT buffer (QIAGEN, Hilden, Germany). Total RNA was extracted using the Rneasy Mini Kit (QIAGEN). First-strand cDNA was synthesized using the T7-(dT)24 primer (Invitrogen). The dsDNA was mixed with T7 RNA polymerase (Invitrogen). Test samples and reference samples were labeled with cyanine-5 (Cy5)- and cyanine-3 (Cy3)-labeled CTP (PerkinElmer, Waltham, MA, USA), respectively. Cy3- or Cy5-labeled cRNA probes were hybridized to the Human 1A ver. 2.0 microarray (Agilent Technologies, Santa Clara, CA, USA), which includes 22,000 genes. The arrays were then scanned as digital image files with GenePix 4000 A (Axon Instruments, Foster City, CA, USA). Relative levels of gene expression were calculated by global normalization. The ratio of Cy3 and Cy5 signal intensity for each spot was quantitatively calculated using GenePix Pro 5.0 (Axon Instruments).

### 4.6. Real-Time PCR

hFOB cells were cultured in six-well culture plates and treated with 3-MC (1 and 10 μM) and β-NF (1 and 10 μM) for three days. AhR antagonist (10 μM CH-223191) was added with the AhR ligands. Real-time PCR was performed using the LightCycler System and the FastStart DNA Master SYBR Green I (Roche Diagnostics, Mannheim, Germany). An initial denaturing step of 95 °C for 10 min was followed by 40 cycles of 10 min at 95 °C; annealing for 15 s at 64 °C (CYP1B1, RPL13A) or 68 °C (aromatase); and extension for 15 s at 72 °C. The mRNA levels in each case were represented as a ratio of RPL13A and evaluated as a ratio (%) compared with each control.

### 4.7. Immunocytochemistry

hFOB cells were seeded on cover glass (Collagen Type I coated Coverglass, φ12 mm, AGC Techno Glass, Shizuoka, Japan) using the culture conditions described above. Cultured discs were fixed with 10% formaldehyde for 10 min. Mouse monoclonal antibodies of aromatase (clone #677) and CYP1B1 (clone 2F8) were obtained from Novartis (Basel, Switzerland) and EMD Millipore (Hayward, CA, USA), respectively. The cells were immunostained using a biotin-streptavidin method with the Histofine kit (Nichirei, Tokyo, Japan).

### 4.8. Cytokine Analysis

hFOB cells were cultured in a 100-mm culture dish. Culture medium was replaced by FBS and phenol red-free medium. 3-MC (10 μM) or β-NF (10 μM) was added, and after 24 h, conditioned medium (total 30 mL) was collected. AhR antagonist (10 μM CH-223191) and 3-MC were simultaneously added to the cell culture medium. Conditioned medium was concentrated to a 5 mL volume using Macrosep Centrifugal Devices (Pall Corporation, Port Washington, NY, USA). In this study, we employed the Human Cytokine Antibody Array 5 (RayBiotech, Inc., Norcross, GA, USA) in order to identify the cytokines released from hFOB cells with/without AhR ligand treatment. The membranes were incubated with biotin-conjugated anti-cytokines (provided with the kit) and developed with horseradish peroxidase-streptavidin and chemiluminescence. Protein dots were visualized with a Las-1000 cooled CCD-camera chemiluminescent image analyzer (Fuji Photo Film, Tokyo, Japan).

### 4.9. Cell Proliferation Assay

Cells were cultured in a 96-well culture plate. Phenol red-free culture medium with 10% charcoal dextran-treated FBS was used. Cells were treated with 3-MC (10 μM) or DMSO (0.005%) for two days, then, testosterone (10 nM) was added for three days. ER blocker (5 μM, ICI 182,780) and aromatase inhibitor (100 nM, Aromatase inhibitor I) were simultaneously added with testosterone (10 nM). AhR antagonist (10 μM CH-223191) was simultaneously added with 3-MC (10 μM).

Cells were then evaluated for cell proliferation using the WST-8 method (Cell Counting Kit-8; Dojindo Inc., Kumamoto, Japan).

### 4.10. Statistical Analysis

Results were expressed as mean ± SD. Statistical analysis was performed with StatView 5.0 J software (SAS Institute Inc., Cary, NC, USA). All data were compared with the vehicle control using paired *t*-tests. A *p*-value < 0.05 was considered statistically significant.

## 5. Conclusions

**Figure 6:**
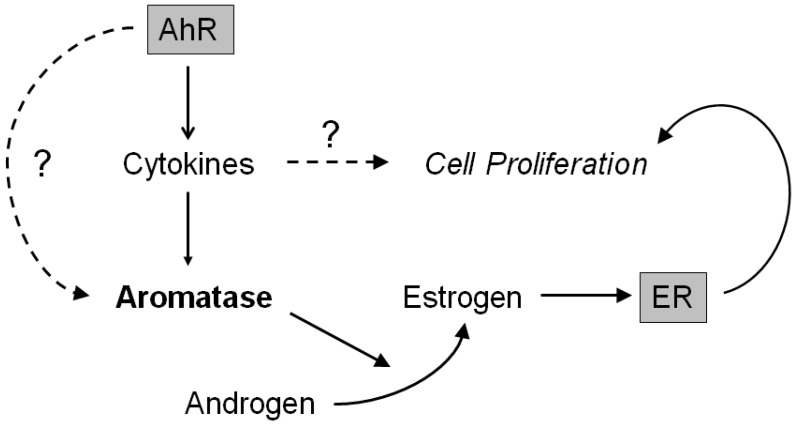
A summary of the proposed AhR pathway in human osteoblasts. AhR, aryl hydrocarbon receptor; ER, estrogen receptor.

The proposed AhR pathway is illustrated in Figure 6. In human osteoblasts, AhR activated by EDCs could possibly induce aromatase expression through the release of cytokines, such as IL-1β and IL-6. Aromatase induced by the EDCs/AhR signal could subsequently exert a cell proliferative effect by locally producing estrogen and then activating ER in osteoblasts. AhR could then directly induce aromatase by binding to its promoter region. In addition, cytokines, including IL-1β and IL-6, could also stimulate osteoblast proliferation. However, in this study, we merely confirmed the phenomenon of the aromatase induction by an AhR agonist, but could not clarify its mechanism through AhR signaling. Therefore, further examinations are required to clarify the effects of EDCs on estrogen action in human bone tissues.

## Figures and Tables

**Table 1:** Gene expression induced by 3-methylcholanthrene treatment in hFOB.

Ratio	Common	Description
23.8	*COL18A1*	Collagen, type XVIII, alpha 1
10.9	*OSTalpha*	Organic solute transporter alpha
7.2	*CABP5*	Calcium binding protein 5
7.1	*GPR34*	G protein-coupled receptor
6.8	*ESR2*	Estrogen receptor 2 (ER beta)
5.2	*COL23A1*	Collagen, type XXIII, alpha 1
4.9	*SLC25A24*	Solute carrier family 25, member 24
4.8	*GRB2*	Growth factor receptor-bound protein 2
4.5	*RUNX1*	Runt-related transcription factor 1
3.7	*IL1F8*	Interleukin 1 family, member 8
3.7	*FGF5*	Fibroblast growth factor 5
3.6	*IL1F7*	Interleukin 1 family, member 7
3.4	*CYP21A2*	Cytochrome P450, family 21, subfamily A, polypeptide 2
3.2	*CYP19A1*	Aromatase
3.2	*OSMR*	Oncostatin M receptor
3.2	*MAPK8IP3*	Mitogen-activated protein kinase 8 interacting protein 3
2.8	*ARSB*	Arylsulfatase B
2.8	*CYP1B1*	Cytochrome P450, family 1, subfamily B, polypeptide 1
2.7	*NR3C2*	Nuclear receptor subfamily 3, group C, member 2
2.7	*SULT1A4*	Sulfotransferase family, cytosolic, 1A, phenol-preferring, member 4
2.5	*CYP4F3*	Cytochrome P450, family 4, subfamily F, polypeptide 3
2.4	*EPHX1*	Epoxide hydrolase 1, microsomal (xenobiotic)
Ratio, a fold change against the vehicle control.		
